# Whole CNS 3D Cryo-Fluorescence Tomography Shows CSF Clearance along Nasal Lymphatics, Spinal Nerves, and Lumbar/Sacral Lymph Nodes

**DOI:** 10.3390/jimaging9020045

**Published:** 2023-02-15

**Authors:** Christian Stokes, Eli F White, Steve Toddes, Nicole Bens, Praveen Kulkarni, Craig F Ferris

**Affiliations:** 1 EMIT Imaging, Baltimore, MD 21201, USA; 2 Center for Translational NeuroImaging, Northeastern University, Boston, MA 02115, USA

**Keywords:** brain clearance, Qdots, nasal turbinates, subarachnoid space, cervical lymphatics, sympathetic ganglia, cervical spinal cord, thoracic spinal cord

## Abstract

Unwanted proteins and metabolic waste in cerebral spinal fluid are cleared from the brain by meningeal and nasal lymphatics and the perineural sheath of cranial nerves; however, the distribution and clearance of cerebral spinal fluid (CSF) along the subarachnoid space of the entire spinal cord is not fully understood. Cryo-fluorescence tomography (CFT) was used to follow the movement of tracers from the ventricular system of the brain down through the meningeal lining of the spinal cord and out to the spinal lymphatic nodes. Isoflurane-anesthetized mice were infused into the lateral cerebroventricle with 5.0 µL of quantum dots [QdotR 605 ITKTM amino (PEG)] over two mins. Mice were allowed to recover (ca 2–3 min) and remained awake and ambulatory for 5, 15, 30, 60, and 120 min after which they were euthanized, and the entire intact body was frozen at −80^°^. The entire mouse was sectioned, and white light and fluorescent images were captured after each slice to produce high resolution three-dimensional volumes. Tracer appeared throughout the ventricular system and central canal of the spinal cord and the entire subarachnoid space of the CNS. A signal could be visualized in the nasal cavity, deep cervical lymph nodes, thoracic lymph nodes, and more superficial submandibular lymph nodes as early as 15 min post infusion. A fluorescent signal could be visualized along the dorsal root ganglia and down the proximal extension of the spinal nerves of the thoracic and lumbar segments at 30 min. There was a significant accumulation of tracer in the lumbar and sacral lymph nodes between 15–60 min. The dense fluorescent signal in the thoracic vertebrae noted at 5- and 15-min post infusion was significantly reduced by 30 min. Indeed, all signals in the spinal cord were ostensibly absent by 120 min, except for trace amounts in the coccyx. The brain still had some residual signal at 120 min. These data show that Qdots with a hydrodynamic diameter of 16–20 nm rapidly clear from the brain of awake mice. These data also clearly demonstrate the rapid distribution and efflux of traces along a major length of the vertebral column and the potential contribution of the spinal cord in the clearance of brain waste.

## 1. Introduction

The subject of cerebral spinal fluid (CSF) clearance, routes, and mechanisms in health and diseases has garnered much attention, particularly with respect to aging and neurodegeneration [1]. How do metabolic waste and unwanted proteins generated at the level of the neurovascular unit find their way out of the interstitial fluid of the surrounding parenchyma? There is evidence that clearance occurs as interstitial fluid moves along paravascular and perivascular routes, accumulating as CSF in the subarachnoid space (SAS) to leave through nasal and meningeal pathways and perineural sheaths [2,3,4]. This clearance is affected by the circadian light–dark cycle as originally reported by Cai and coworkers using magnetic resonance imaging (MRI) to follow the circulation and brain penetrance of gadolinium tracer infused into the cerebral ventricle in awake rats during the scanning session [5]. Brain clearance is most pronounced during periods of rest and sleep and is reduced during the waking hours of the light–dark cycle [6,7].

It is well documented that CSF drains from the SAS through the cribriform plate along olfactory nerves to the nasal mucosa lining the nasal turbinates and from there through nasal lymphatics to the deep cervical lymph nodes [8,9,10]. There is also a direct route of CSF clearance to the deep cervical lymph nodes via lymphatic vessels located in the meninges along the ventral surface of the brain in mice [11,12]. While less emphasized, there is evidence from early studies using horse radish peroxidase (HRP) of tracers appearing in the lumen of cerebral blood vessels, suggesting a direct clearance of brain CSF into the general circulation [13,14]. This finding of movement of tracer from CSF directly into the blood circulation was advanced by Lam et al. at the level of the spinal cord [15]. Indeed, the spinal cord itself is reported to have a significant role in the clearance of CSF via meningeal lymphatics and perineuronal sheaths [16,17].

Recently, Leaston et al. reported a novel pathway by which waste from the brain moves to the nasal mucosa and then to the nasal pharynx to ultimately be swallowed [18]. This finding was confirmed in two studies, one using MRI to follow ferumoxytol circulation and clearance from the brain in awake rats during the imaging session, and a second using Qdot fluorescence microscopy of ex vivo samples of esophagus. Within 10–15 min after intracerebroventricular (ICV) infusion, tracer from both studies can been seen outside the brain along the nasopharynx and esophagus. Indeed, this finding was the motivation behind these studies in mice. Using 3D cryo-fluorescent tomography (CFT) we hoped to see the accumulation over time of ICV-administered Qdots in the stomach and intestine of mice. Unfortunately, due to the background autofluorescence, particularly in the gastrointestinal tract, this was not possible. Instead, we were able to create a time series showing the distribution, localization, and clearance of Qdot fluorescence over the entire brain and spinal cord and associated lymphatic nodes. These unexpected data are presented and discussed with an emphasis on the spinal cord as a route for CSF clearance.

## 2. Materials and Methods

### 2.1. Animal Usage

Male C57BL/J6 mice (n = 5) approximately 100 days old and weighing between 28–30 gm were obtained from Charles River Laboratories (Wilmington, MA, USA). Mice were maintained on a 12:12 h light–dark cycle with lights on at 07:00 h and allowed access to food and water ad libitum. All mice were acquired and cared for in accordance with the guidelines published in the Guide for the Care and Use of Laboratory Animals (National Institutes of Health Publications No. 85–23, Revised 1985) and we adhered to the National Institutes of Health and the American Association for Laboratory Animal Science guidelines. The protocols used in this study complied with the regulations of the Institutional Animal Care and Use Committee at the Northeastern University and adhered to the ARRIVE guidelines for reporting in vivo experiments in animal research [19].

### 2.2. Quantum Dot Infusion

Quantum dots [QdotR 605 ITKTM amino (PEG)] with a hydrodynamic diameter of 16–20 nm were obtained from Thermo Fisher Scientific (Waltham MA, USA). Qdots were diluted 1/100 in sterile saline prior to infusion. While under isoflurane anesthesia (unresponsive to foot pinch, 40–45 breathes/min) the skin was incised and the skull over bregma exposed. A 30-gauge needle connected to a 200 µL syringe was directed toward the lateral cerebroventricle (stereotaxic coordinates of burr hole: 0.50 mm rostral to bregma, 1.0 mm lateral to midsagittal suture, and 3.5 mm down from the skull surface). Five µL of Qdots were infused over 2 min. Mice were treated with buprenorphine (1.5 mg/kg) and the open skin was repaired with surgical glue. Mice were allowed to recover from anesthesia and were ambulatory within 2–3 min. At time intervals of 5-, 15-, 30-, 60-, and 120-min post recovery, mice were sacrificed with Euthasol^®^ (150 mg/kg, IP) and the entire body was laid out in a prone position on aluminum foil and placed in a −80^0^ refrigerator. These specimens were then shipped frozen on dry ice to Emit Imaging for sectioning and analysis (Baltimore, MD, USA).

### 2.3. Cryo-Fluorescence Tomography (CFT)

The mice were imaged on Emit Imaging’s Xerra platform. The Xerra is an automated CFT platform with an integrated cryomicrotome and proprietary software that captures 2D white light and fluorescent images of ex vivo serial sections and compiles them into 3D images. Sample preparation involved freezing 5 mice onto a single block of O.C.T (Ultrafreeze OCT Clear, Cancer Diagnostics Durham, NC, USA). The block with a field of view of 17 cm × 10 cm was then placed in the Xerra and the mice were then automatically sectioned in 35 µm slices over 17 h at −15 °C. The newly exposed block face at each section was imaged for white light and fluorescence signal, while the shaved tissue was discarded. Sixteen-bit fluorescence images were acquired with consistent exposure times of 5 ms, 50 ms, 500 ms, 1500 ms, and 2500 ms. These images were then combined into a single 32-bit high dynamic range image to extend the Xerra’s detection limits beyond a single exposure.

## 3. Results

**Figure 1:**
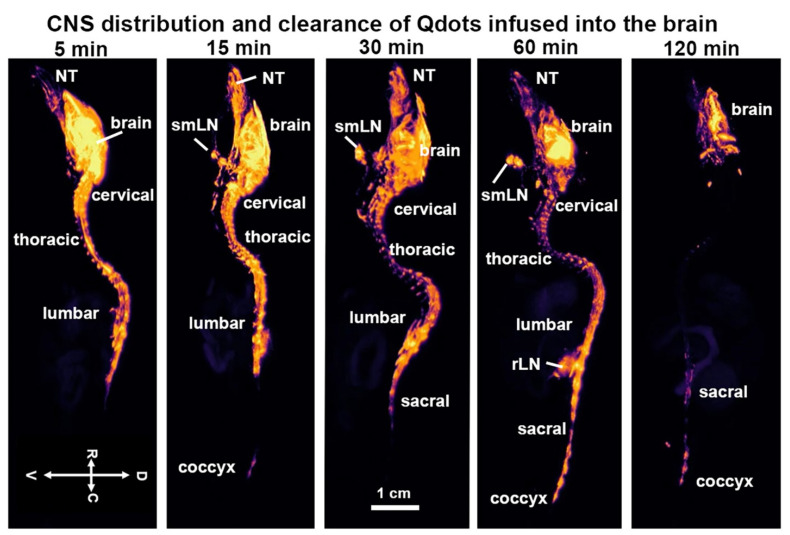
CNS distribution and clearance time series. Shown are cryo-fluorescent microscopic images of the CNS taken at different time intervals following infusion into the lateral cerebroventricle. Highlighted are the different segments of the vertebral column, e.g., cervical, thoracic, lumbar, sacral, and coccyx. Abbreviations: NT—nasoturbinates; rLN—renal lymph nodes; smLN—submandibular lymph nodes. Scale bar 1 cm.

**Figure 2:**
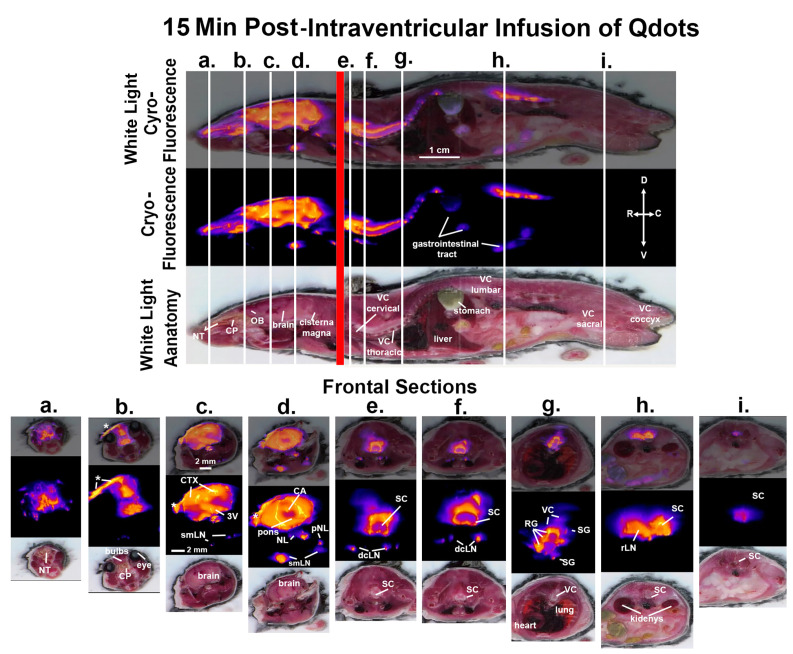
CNS distribution and clearance at 15 min. Shown above are sagittal sections depicting the same mouse imaged for white light anatomy, cryo-fluorescence, and both together at 15 min post infusion of ICV Qdots. The head of the mouse and the body were not in plane, hence the red line demarking the intersection of the separate images. The perpendicular white lines labeled (**a.**–**i.**) denote the position of the frontal sections shown below. Abbreviations: NT—nasoturbinates; CP—cribriform plate; CTX—cortex; 3V—third ventricle; smLN—submandibular lymphatic nodes; CA—cerebral aqueduct; NL—nasal lymphatics; pNL—parotid lymphatic nodes; SC—cervical spinal cord; dcLN—deep cervical lymph nodes; VC—thoracic vertebra; RG—root ganglia; SG—sympathetic ganglia; rLN—renal lymph nodes. Scale bar for sagittal sections is 1 cm. Scale bar for frontal sections is 2 mm.

**Figure 3:**
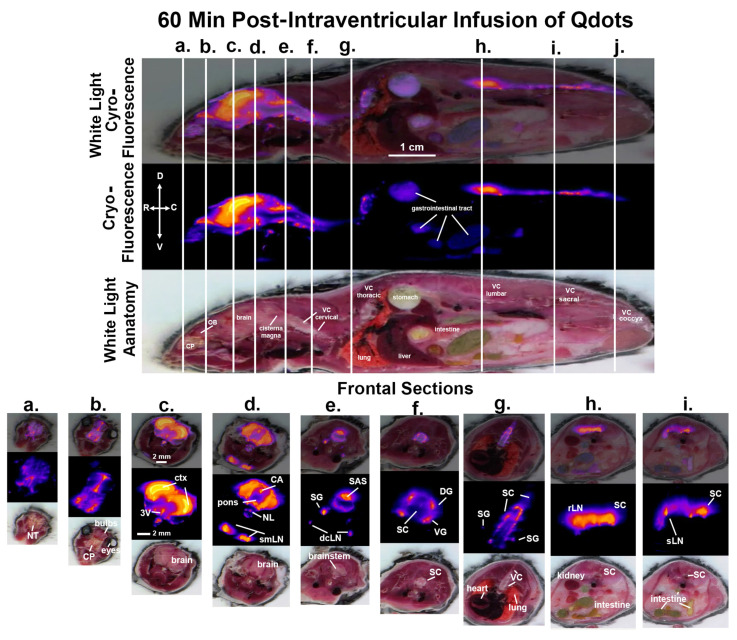
CNS distribution and clearance at 60 min. Shown above are sagittal sections depicting the same mouse imaged for white light anatomy, cryo-fluorescence, and both together at 60 min post infusion of ICV Qdots. The perpendicular white lines labeled (**a.**–**i.**) denote the position of the frontal sections shown below. Abbreviations are the same as in Figure 2. OB—olfactory bulbs; SAS—subarachnoid space; DG—dorsal root ganglia; VG—ventral root ganglia: laLN—aortic lymph nodes. Scale bar 1 cm and 2 mm sagittal and horizontal sections, respectively.

Shown in Figure 1 are CFT images of the CNS taken at different time intervals following recovery from the infusion of Qdots into the lateral cerebroventricle. Note images from all subjects were collected simultaneously from the same block. Highlighted are the different segments of the vertebral column, e.g., cervical, thoracic, lumbar, sacral, and coccyx. The distribution of fluorescence across much of the vertebral column occurred within five minutes of ICV infusion. Fluorescence can be observed in the most rostral part of the image, in what is the nose of the mouse, filling the nasoturbinates (NT). At 15 min, a signal can also be seen in the coccyx, which is at the end of the vertebral column (see Figure 2). The submandibular lymph nodes (smLN) accumulate Qdots at 15 min. The signal persists for up to 60 min but is absent by 120 min. By 60 min, the entire vertebral column is heavily penetrated by Qdots (see Figure 3) which are all essentially absent by 120 min.

Shown in Figure 2 are sagittal sections taken 15 min post infusion of Qdots depicting the same mouse imaged for white light and cryo-fluorescence. The head of the mouse and the body were not in plane, hence the red line marking the intersection of the separate images. The perpendicular white lines labeled (a.–i.) denote the position of the frontal sections shown below. Section (a.) shows a fluorescent signal in the nose of the mouse localized to the nasoturbinates (NT). Section (b.) shows a signal at the level of the olfactory bulbs and the underlying cribriform plate (CP). The asterisk identifies an accumulation of Qdots along the surface of the skull that leaked from the infusion needle. Section (c.) depicts a signal throughout the brain, particularly concentrated in the cortex (CTX) and along the third ventricle (3V). A fluorescence signal is also starting to appear in the submandibular lymphatic nodes (smLN). Section (d.) shows a signal in the brain at the level of the pons. The cerebral aqueduct (CA) is highlighted along with a signal in the smLN and what could be identified as the nasal lymphatics (NL) and putative parotid lymphatic nodes (pNL). Section (e.) shows a section of the cervical spinal cord (SC) and the underlying deep cervical lymph nodes (dcLN). Section (f.) shows a similar image of the cervical SC just rostral to thoracic cavity. Section (g.) shows several thoracic vertebrae (VC) and signal intensity along the root ganglia (RG) and smaller areas of signal accumulation in what would be the putative sympathetic ganglia (SG). Section (h.) shows the lumbar spinal cord and the adjacent renal lymph nodes (rLN) identified by their proximity to the kidneys as shown below in the light field anatomy. The most caudal Section (i.) depicts the signal in the spinal cord coccygeal region of the vertebral column.

Shown in Figure 3 are sagittal sections taken 60 min post infusion of Qdots depicting the same mouse imaged for white light anatomy and cryo-fluorescence. The cryo-fluorescent image shows autofluorescence associated with the gastrointestinal tract. The perpendicular white lines labeled (a.–i.) denote the position of the frontal sections shown below. Section (a.) shows fluorescent signal in the nose of the mouse localized to the nasoturbinates. Section (b.) shows a signal at the level of the olfactory bulbs (OB) and the underlying cribriform plate. Section (c.) depicts signal intensity localized primarily to the cortex of the brain and along the third ventricle (3V). Section (d.) shows a signal in the brain at the level of the pons with the highest intensity in the cortex and cerebral aqueduct. Below are the submandibular lymph nodes and the putative nasal lymphatic vessels (NV). Section (e.) shows the brainstem and overlying subarachnoid space (SAS). The small signal intensity comes from what we interpret as the putative sympathetic ganglia and below that are the deep cervical lymph nodes. Section (f.) shows the cervical spinal cord proximal to the thoracic cavity. The four points of signal intensity are the putative dorsal (DG) and ventral (VG) ganglia. Section (g.) shows several thoracic vertebrae and the spinal cord. The small points of signal intensity are the putative sympathetic ganglia (SG). Section (h.) shows the lumbar spinal cord and the adjacent renal lymph nodes. The caudal Section (i.) depicts the signal in the spinal cord and the adjacent sacral lymph nodes (sLN). Section (j.) shows the coccygeal region of the vertebral column.

**Figure 4:**
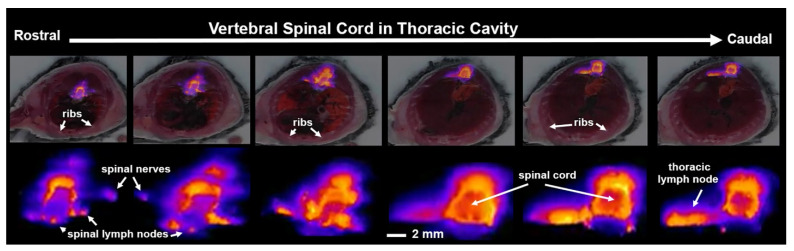
Distribution and clearance along the thoracic spinal cord. Shown above are serial frontal sections of white light anatomy and cryo-fluorescence at the level of the vertebral spinal cord acquired 15 min post infusion of Qdots ICV (scale magnified images of the fluorescent signal at each segment of the spinal cord are shown below (scale bar 2 mm).

Shown in Figure 4 are fluorescence images taken of t**he** thoracic spinal cord 15 min post ICV infusion of Qdots. The images highlight not only the rapid distribution of the tracer down the subarachnoid space, but its egress into the putative spinal lymphatic nodes, spinal nerves, and thoracic lymph nodes.

## 4. Discussion

These studies using CFT enabled us to follow the distribution, localization, and clearance of Qdots over the entire CNS. Cifuentes and coworkers provided clear evidence in rats of the rapid movement of CSF from the lateral cerebroventricle down the central canal of the spinal cord. By 20 min after intraventricular infusion, HRP tracer reached a maximum across all segments of the spinal cord that decreased sharply by one hour and was cleared by two hours [13]. In a comprehensive study, Ma and coworkers used near-infrared and gadolinium-based MRI contrast agents infused ICV in anesthetized and awake mice and reported movement down the central canal and subarachnoid space of the spinal cord to leave predominantly at the sacral level through lymphatic vessels leading to the sacral/iliac lymph nodes [16]. In their study, the distribution of fluorescent tracer at the level of the thoracic spinal cord was apparent by 30 min in awake mice but delayed with anesthesia. The confound of anesthesia when studying brain clearance was also noted by Gakuba et al. using near-infrared fluorescence imaging and contrast enhanced MRI to follow the distribution of tracers injected into the cisterna magna in awake mice and compared these results with anesthesia. Contrast agent rapidly spread across the brain when mice are awake but was severely limited with anesthesia [20].

The application of awake MRI to follow brain clearance has been used in rats to study the effect of the circadian light–dark cycle [5]. During the period of rest and sleep (light phase in the rodent light–dark cycle) there is a greater efflux in tracer-injected ICV compared with rats imaged during the dark or active phase of the light–dark cycle. The distribution of tracer across the CNS during the light phase and its efflux occurs within 30 min of injection. These results in rats using MRI to follow clearance are similar to the data generated in this study using CFT. The fluorescent tracer is rapidly distributed and cleared while mice are aroused and awake during the light phase of their circadian cycle when they should be sleeping. Sleep and rest are a critical period in the circadian cycle helping to promote brain clearance [6,7,21,22,23]. The clearance linked to the circadian sleep–wake cycle is judged to be essential in the removal of unwanted metabolic waste and proteins that could be contributing to Alzheimer’s, Parkinson’s, and dementia associated with aging [22,24]. It has also been proposed that the flow of CSF and parenchymal clearance along the perivascular system is influenced by circadian changes in brain temperature and blood flow at the level of the microvasculature [25]. Brain temperature is a circadian rhythm entrained by the light–dark cycle [26]. The circadian change in temperature affects the timing of sleep [27,28], e.g., rising in the morning before awakening and lowering in the evening before the onset of sleep. The temperature of the brain is higher than the body temperature and heterogeneous as some brain areas are cooler than others [29,30,31]. An increase in blood flow to metabolically active areas is necessary to buffer the higher temperatures but has the unwanted effect of increasing resistance to perivascular clearance [25]. Given the large surface to volume ratio of the spinal cord, the temperature may be closer to body temperature, minimizing the need for the obligatory increase in blood flow that would impair clearance. Thus, over the circadian sleep–waking cycle the spinal cord may be more efficient at removing CNS waste than the brain.

In the present study using CFT with Qdots, the distribution of fluorescence around the thoracic spinal cord and the associated spinal lymphatic nodes and nerves could be viewed as early as 15 min post ICV infusion (see Figure 4). The rapid distribution and clearance across the entire CNS could be explained in part by the chemistry of Qdots and experimental conditions, i.e., mice were studied during the light phase of the light–dark cycle and while fully awake as noted above. The rapid appearance of Qdots in the submandibular lymph nodes corroborates an earlier study by Mathieu et al. using Qdots with an emission spectrum of 655 and a hydrodynamic diameter ca. 19 nm, similar to that used in our study [32]. Infusion of 3 µL of Qdots into the cisterna magna of mice following in vivo hyperspectral imaging showed fluorescence signal in the submandibular lymph nodes as early as 20 min with maximum fluorescence by 40 min. Interestingly, this in vivo imaging study showed preferential clearance to the submandibular lymph nodes and not the deep cervical lymph nodes highlighted by other studies [33]. This finding may be due to the size and chemistry of the Qdots that favor this route of clearance. Using near-infrared fluorescence imaging in awake mice, Ma et al. showed rapid accumulation in the submandibular lymph nodes peaking at 30 min and rapidly decreasing by 60–90 min [34]. This submandibular route of CSF clearance has been attributed to clearance along the optic nerves and orbital connective tissue.

Our study shows a rapid global distribution and sustained localization of fluorescence signal over the entire brain and spinal cord that peaks as early as 30 min post ICV infusion but is almost eliminated by 120 min. This raises an important question that has been addressed at the level of the brain but not fully considered across the entire spinal cord—how does it get out? This question was addressed by Liu et al. by looking at the distribution of fluorescent tracer infused into white and gray matter at the level of the thoracic spinal cord in rats [35]. Tracer accumulated along the microvasculature, i.e., arterioles, capillaries, and venules, adding the spinal cord to a body of literature that clearance of waste from interstitial fluid is carried along paravascular/perivascular routes. Waste from these microvascular routes can mix with the CSF in the meninges surrounding the cord and be cleared via spinal lymphatic vessels [36,37,38]. Indeed, we were able to visualize putative lymphatic nodes along the spinal cord in the cervical and thoracic sections with CFT. Studies in humans report CSF flow along the lumbar nerves following intrathecal injection of tracers into the subarachnoid space [39,40]. Bechter et al. reported the rate of CSF flow to be 10 cm/h, noting the outflow from the lumbar spinal cord was remarkable [40].

Images collected with CFT across the entire CNS were not able to provide the required resolution to clearly identify lymphatic vessels, nodes, nerves, and ganglia (see Figure 4) associated with the spinal cord as reported in other publications using different fluorescence microscopic procedures and histological preparations [16,41]. For example, iDISCO with light sheet fluorescence microscopy (iDISCO/LSFM) provides exquisitely detailed 3D reconstructions of lymphatic vessels associated with the cervical/thoracic spinal cord of mice. However, the imaging modality is limited to samples of less than 1.5 cm^3^ and thereby unable to capture the whole CNS transport and clearance as shown here [38].

## Data Availability

All of the data is provided in the manuscript.
